# Analysis of the Tensile Failure Process of TRC Based on Load–Displacement Curves and DIC Full-Field Strain: Multi-Crack Stable Development Mechanism Under the ACK Framework

**DOI:** 10.3390/ma19153279

**Published:** 2026-08-03

**Authors:** Feiting Shi, Shuangcheng Wu, Li Li, Haoyu Huang, Zhengdong Zhi, Yuan Hou, Yifei Yang, Peng Cao

**Affiliations:** 1 School of Civil Engineering, Yancheng Institute of Technology, Yancheng 224051, China; shifeiting@ycit.cn (F.S.); 19850970287@163.com (S.W.); 19715158305@163.com (L.L.); huanghaoyu0920@163.com (H.H.); zhengdongzhi@163.com (Z.Z.); houy487786@163.com (Y.H.); 17839613163@163.com (Y.Y.); 2 Jiangsu Engineering and Research Center of Intelligent Disaster Prevention in Coastal Transportation Infrastructure, Yancheng 224051, China; 3 College of Architecture and Civil Engineering, Beijing University of Technology, Beijing 100124, China

**Keywords:** textile-reinforced concrete, carbon fiber, uniaxial tension, failure mechanism

## Abstract

This study systematically investigates the effects of reinforcement ratio, textile orientation, and interface treatment on the tensile behavior of carbon fiber textile-reinforced concrete (TRC) through uniaxial tensile tests and numerical simulation. The experimental results indicate that as the reinforcement ratio increases from 0.44% to 1.33%, the ultimate tensile stress rises from 1.86 MPa to 4.08 MPa (an increase of 119%), the ultimate tensile strain increases from 0.86% to 1.72% (an increase of 100%), and the average crack spacing decreases from 24.3 mm to 8.7 mm (a reduction of 64%). Correspondingly, the failure mode transitions from brittle single-crack fracture to multi-crack quasi-ductile failure. Compared with transverse orientation, the longitudinal orientation yields a 32.7% increase in ultimate tensile stress and a 46.2% increase in ultimate tensile strain, primarily attributable to a 35–45% enhancement in interfacial frictional stress. Under the longitudinal configuration, carbon-glue interfacial treatment further improves the ultimate tensile stress by 36.9% and the ultimate tensile strain by 42.1%, while reducing the average crack spacing to 8.1 mm and shifting the failure mode toward fiber rupture-dominated failure. Digital image correlation (DIC) analysis reveals that the strain inhomogeneity index decreases from 0.65–0.78 to 0.28–0.35. The finite element simulation results indicate that the error in peak load remains below 5%, and the error in crack spacing remains below 10%. This study establishes a quantitative regulatory framework encompassing reinforcement ratio, textile orientation, and interface treatment, thereby providing a basis for performance prediction and optimization of TRC.

## 1. Introduction

Fiber-reinforced composites have been increasingly used in the construction industry because of their high strength-to-weight ratio, corrosion resistance, and potential for improving the durability and service performance of structural components [1]. Against this backdrop, Textile Reinforced Cementitious Composites (TRC), as a novel composite material that replaces traditional steel reinforcement with continuous fiber textiles, has gradually emerged as a significant research direction in the field of high-performance cementitious materials in recent years. TRC forms a collaborative load-bearing system by embedding continuous fiber textiles—such as alkali-resistant glass fibers, carbon fibers, or basalt fibers—into a fine-aggregate mortar or cementitious matrix. This configuration ensures load-bearing capacity while substantially reducing the thickness of the protective cover, thereby enabling the realization of thin-walled and lightweight structural components [2]. Owing to the corrosion resistance, fine diameter, and flexible arrangement according to stress demands of textile fibers, TRC members can effectively inhibit crack propagation and control crack width under tensile and flexural loading, thus significantly enhancing structural durability [3]. Compared with conventional reinforced concrete, TRC effectively avoids performance deterioration caused by steel corrosion, demonstrating considerable engineering potential in the rehabilitation and strengthening of existing structures and in the fabrication of novel thin-walled precast components [4]. For instance, experimental research by Promis et al. [5] indicated that strengthening reinforced concrete beams with textile-reinforced cementitious composites could significantly improve their ultimate load-bearing capacity and deformability without substantially increasing self-weight or cross-sectional dimensions, thereby verifying the feasibility and advantages of TRC in engineering applications.

Among the various mechanical properties of TRC, tensile behavior is generally regarded as one of the most representative fundamental properties. This is because the engineering advantages of TRC are ultimately epitomized by its post-cracking load-bearing capacity and crack control performance in the tensile zone. Extensive research has demonstrated that, under uniaxial tension, TRC and related textile-reinforced cementitious composites exhibit a stress–strain response markedly different from that of ordinary brittle cementitious materials. the specimen initially displays approximately linear elastic behavior until the onset of matrix cracking; subsequently, as loading continues, the stress can be maintained or even increase after the first crack, with multiple cracks progressively developing within the gauge length, leading to a pronounced strain-hardening stage [6]. During the strain-hardening stage, multiple cracks develop in a relatively uniform manner, with crack spacing and crack width effectively constrained to low levels due to the bridging action of the fiber textile [7]. As the number of cracks increases, tensile stress is gradually transferred from the matrix to the fiber bundles. Although the overall stiffness of the composite degrades, stable load-bearing capacity is still sustained. As loading approaches the ultimate state, the principal tensile stress is primarily undertaken by the fiber bundles, and failure eventually occurs due to fiber rupture or fiber-matrix interfacial debonding [8]. The aforementioned “multi-crack strain-hardening” tensile response characteristics are mechanistically consistent with the multiple cracking theory proposed by Aveston and Kelly [9] and constitute a key feature distinguishing TRC from conventional reinforced concrete and short fiber reinforced concrete. A large body of experimental results further indicates that, provided the effective reinforcement ratio of the fiber textile exceeds a critical value and appropriate interfacial bonding conditions are met, TRC can sustain stable post-cracking load-bearing capacity and form multiple uniformly distributed fine cracks, thereby exhibiting tensile hardening behavior analogous to that of metallic materials. Peled and Bentur [10], through uniaxial tensile tests on TRC specimens with different textile architectures, found that the introduction of textile grids enabled the formation of more than eight mesoscopic cracks, with the ultimate tensile strain exceeding that of the plain matrix by more than one order of magnitude. Mobasher et al. [11], employing an analytical approach to simulate the entire tensile process of TRC, successfully reproduced the multi-crack development and associated stiffness degradation, providing theoretical support for understanding the macroscopic tensile response of TRC. More recently, DIC-based automated crack evaluation has enabled quantitative characterization of crack distribution, crack width, and full-field deformation in TRC [12], while advanced numerical modeling of TRC systems has further integrated direct tensile tests, DIC-based image analysis, micro-computed tomography, and CDP-based finite element modeling to reveal cracking evolution, interfacial effects, and thermomechanical damage mechanisms [13]. In summary, the typical tensile strength of TRC generally ranges from 5 to 15 MPa, while the ultimate tensile strain reaches a stable level between 0.5% and 1.5%, significantly exceeding the fracture strain level of ordinary concrete (approximately 0.01%) [14,15].

Although the basic characteristics of the tensile behavior of TRC have been widely recognized, its mechanical response is influenced by a variety of factors, often exhibiting significant coupling effects among different parameters. Among these, the reinforcement ratio of the fiber textile and its configuration in the direction of loading are considered critical parameters influencing tensile behavior [16]. Previous studies have revealed a distinct critical value for the volume fraction of warp fibers (parallel to the loading direction): below this critical value, the material experiences a stress drop upon first cracking, forming only a single dominant crack and failing rapidly; above this threshold, the material exhibits a strain-hardening response accompanied by stable multi-crack development. As the fiber volume fraction increases, the number of cracks per unit length increases and the crack spacing decreases, thereby significantly enhancing the overall ductility and post-cracking residual load-bearing capacity. Experimental results reported by Zhu et al. [17] indicated that when the warp fiber volume fraction was increased from 0.24% to 1.09%, the number of cracks increased from 2–3 to 7–8, the average crack spacing decreased from approximately 60 mm to 20 mm, and the peak strain and tensile toughness were substantially improved. In addition to the reinforcement ratio, the geometric parameters of the textile architecture also exert a significant influence on the tensile response of TRC. Recent studies on textile structure optimization further indicate that textile straightening, three-dimensional architecture, epoxy impregnation, and reinforcement volume fraction can significantly affect post-cracking stiffness, crack distribution, toughness, and reinforcement utilization efficiency in TRC composites [18]. A comparative study by Paul et al. [19] further demonstrated that, under identical fiber content, textiles with a yarn spacing of 16.5 mm increased the first-cracking load by approximately 40% and promoted a transition from strain-softening to strain-hardening tensile response. Moreover, weaving patterns, surface coating treatment, and differences in fiber type also significantly affect the tensile behavior by altering the effective participation rate of fibers and the interfacial load transfer pathways [20,21]. The fiber–matrix interfacial behavior has also been shown to be closely related to textile configuration and matrix composition, since changes in textile geometry can alter interfacial degradation, slippage, and post-cracking damage evolution in carbon-fiber TRC systems [22].

Previous studies have advanced the understanding of fiber- and textile-reinforced cementitious composites from different perspectives, including microcracking behavior, mechanical performance, durability, and thin-plate structural response [23,24,25]. However, most existing research remains focused on parametric evaluations of reinforcement ratio, textile configuration, and interfacial treatment, while the intrinsic coupling mechanism governing crack initiation, interfacial slip, and strain localization has not been fully established under a unified theoretical–numerical framework. First, previous studies have extensively examined the effects of reinforcement ratio, textile architecture, and surface or interfacial treatment, but these factors have often been investigated separately or through limited parameter combinations. As a result, the coupled influence of reinforcement ratio, textile orientation, and interfacial load transfer on the transition from first cracking to saturated multiple cracking remains insufficiently clarified. Second, most existing studies have interpreted the tensile response mainly from macroscopic stress–strain or load–displacement curves, toughness indices, or average crack parameters [26,27], whereas the direct correspondence between the characteristic sawtooth fluctuations in these curves and the underlying processes of crack initiation, crack propagation, interfacial slip, and strain localization has not been systematically established. Third, although the ACK theory provides an important basis for describing multiple cracking in fiber-reinforced cementitious composites, its integration with DIC-based full-field strain measurements and finite element simulation remains limited, particularly for carbon-fiber TRC systems with different reinforcement ratios, textile configurations, and interfacial treatments [7,28,29]. Therefore, a more integrated experimental–numerical framework is still required to connect macroscopic tensile behavior, full-field strain evolution, interfacial load transfer, and the stable development mechanism of multiple cracks.

To address these limitations, this study develops a mechanism-oriented and modeling framework-based investigation of the tensile behavior of carbon-fiber TRC. Unlike previous studies that mainly focus on isolated parameter effects, the present work integrates the Aveston–Kelly (ACK) analytical theory with a finite element modeling framework incorporating explicit interface bond–slip behavior. The proposed framework enables a direct linkage between macroscopic load–displacement response and micro-scale crack evolution, including crack initiation, propagation, and interfacial debonding. In addition, digital image correlation (DIC) full-field strain measurements are employed to experimentally capture strain localization and validate the numerically predicted crack development process. Furthermore, the interfacial parameters are calibrated based on single-fiber pullout tests, allowing a physically consistent representation of load transfer and residual frictional resistance. This multi-scale integrated approach provides a unified understanding of TRC tensile behavior from theoretical, numerical, and experimental perspectives. Overall, the novelty of this study lies in transforming a conventional parameter-based TRC investigation into a mechanism-driven multiscale framework that unifies ACK theory, interface mechanics, and full-field experimental–numerical validation of crack evolution in carbon-fiber TRC.

## 2. Experimental Program

### 2.1. Experimental Materials and Preparation

**Figure 1:**
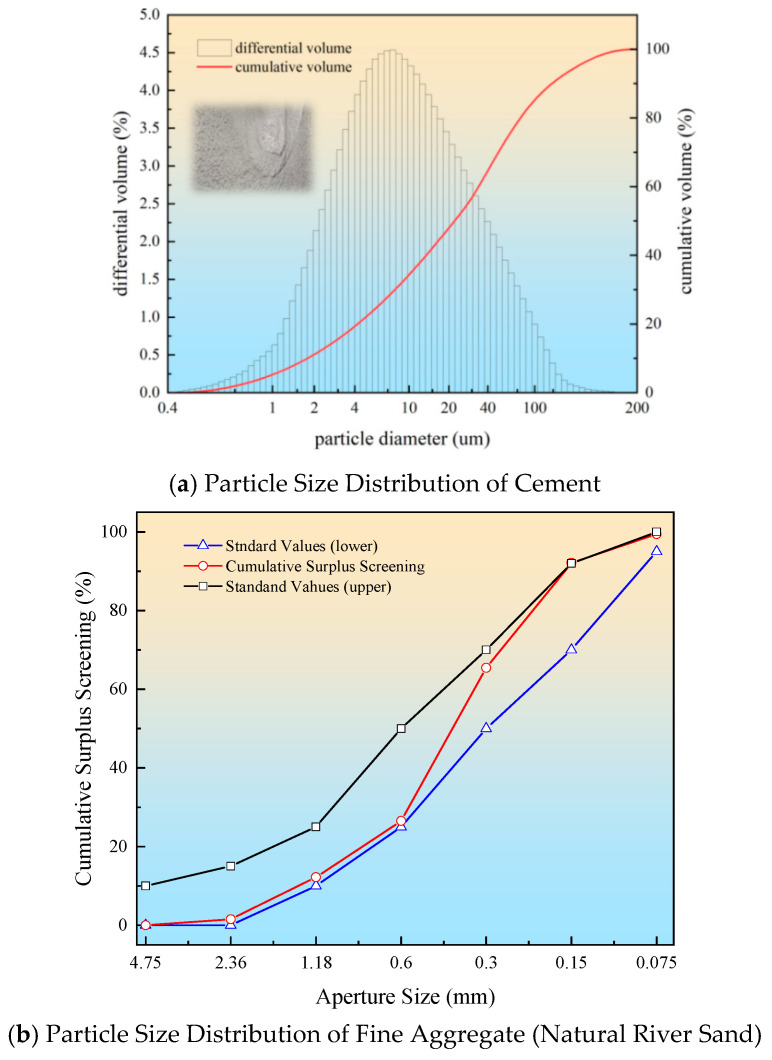
Physical properties of raw materials.

Figure 1 presents the physical appearance of the raw materials used for the preparation of the TRC specimens. The matrix materials adopted in the experiment include cementitious materials, aggregates and chemical admixtures. Among them, the cementitious material is ordinary Portland Cement (P.O 42.5); the fine aggregate is natural river sand with a maximum particle size of 1.2 mm and a fineness modulus of 1.8; marble powder with a particle size distribution (D50) of 45 μm is additionally incorporated as a functional filler. To regulate the properties of fresh mortar, the following admixtures are added: UEA expansive agent, lithium carbonate accelerator and hydroxypropyl methyl cellulose (HPMC) water retention agent. All components are mixed with tap water. The mix proportion design of the matrix is shown in Table 1, and the chemical composition of ordinary Portland Cement is listed in Table 2.

The processing procedure for concrete is as follows: First, the fine aggregate and cement were placed into the mixer according to the mix proportions listed in Table 1 and mixed together. Then, water was slowly added while stirring thoroughly. The mixed concrete was subsequently poured into molds with dimensions of 100 mm × 100 mm × 100 mm, and the molds were finally placed on a vibrating table for compaction. After 24 h of curing, the molds were removed, and the plain concrete specimens were transferred to a standard curing room, where they were cured at 20 ± 3 °C under controlled humidity conditions for 28 days to ensure strength development.

**Figure 2:**
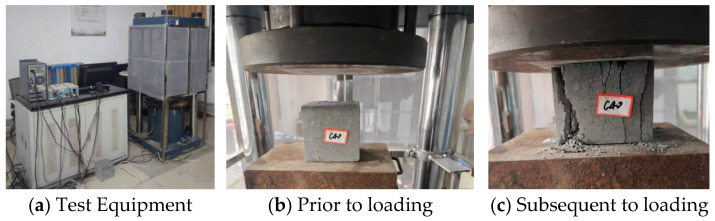
Compressive Strength Test of Matrix Concrete Specimens.

To characterize the mechanical properties of the matrix material, 100 mm × 100 mm × 100 mm cubic specimens were prepared for compressive strength testing. Loading tests were conducted at curing ages of 7 d, 14 d, 21 d, and 28 d, as illustrated in Figure 2. The test results showed that the compressive strength of the matrix increased with curing age, reaching 33 MPa, 42 MPa, 45 MPa, and 50 MPa, respectively. The 28-day compressive strength reached 50 MPa, providing a matrix strength basis for subsequent tensile tests and modeling.

**Figure 3:**
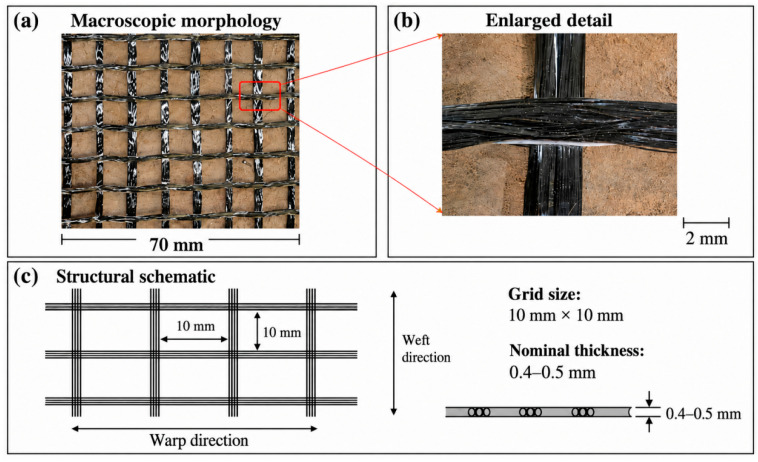
Carbon Fiber Grid Cloth.

The fabric reinforcement used was a carbon fiber mesh (grid) with a nominal thickness of 0.4–0.5 mm and a grid size of 10 mm × 10 mm. Its macroscopic morphology is shown in Figure 3, and the specific performance parameters are listed in Table 3. To investigate the influence of the weaving method on the tensile properties of the material, mesh sheets with different configurations were prepared by cutting and retaining fiber bundles oriented in specific directions (warp or weft), as illustrated in Figure 3. Subsequently, corresponding TRC tensile specimens were fabricated using these mesh sheets.

In the specimen preparation process, the reinforcement ratio was adopted as the key variable parameter. Carbon fiber textile mesh was used as the reinforcing material, and the specimens were fabricated using a direct mold-casting method. The reinforcement ratio was defined as the ratio of the cross-sectional area of the fibers in the reinforcement direction (A*_f_*) to the total cross-sectional area of the tensile zone of the specimen (A*_f_*/A). Accordingly, the reinforcement ratios for specimens with single-layer, double-layer, and triple-layer carbon fiber mesh were 0.44%, 0.88%, and 1.33%, respectively.

**Figure 4:**
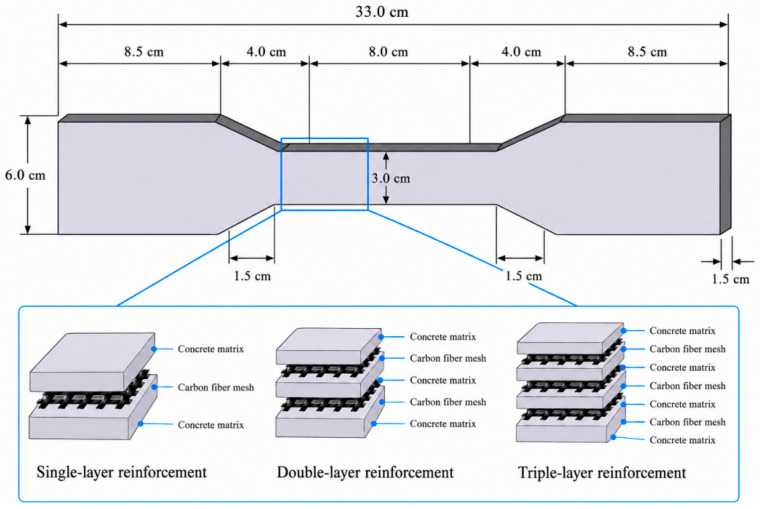
Fiber Web Arrangement in the Stress Zone.

During the mold preparation stage, the carbon fiber textile mesh was first fixed in the designated positions within a custom mold, as shown in Figure 4. To ensure the stability of the mesh during the casting process, a pre-curing treatment was applied to the fiber mesh using carbon glue and epoxy resin. This procedure effectively enhanced the stiffness of the fabric, preventing deformation and displacement during subsequent construction steps. For specimens with different numbers of layers, the single-layer mesh was placed at the center of the specimen. For the double-layer and triple-layer specimens, the interlayer spacings were controlled at 4 mm and 2 mm, respectively, while the cover thickness was maintained at 3 mm for all specimens.

**Figure 5:**
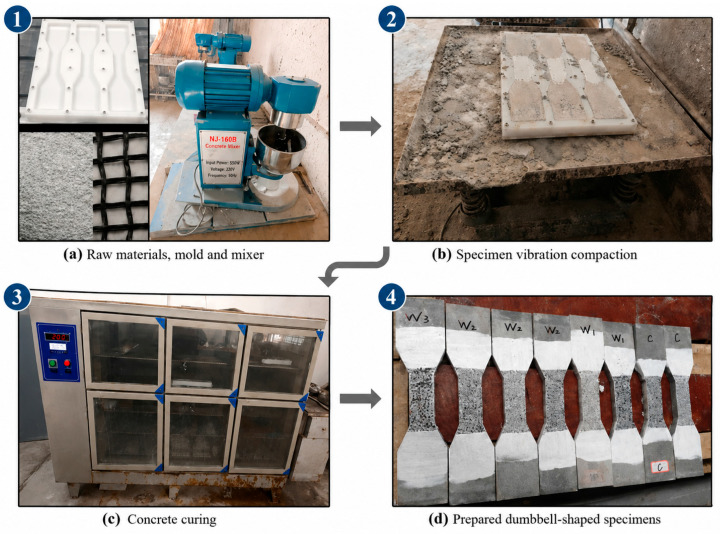
Experimental Flowchart.

During the material preparation stage, each constituent material was accurately weighed according to the predetermined mix proportion, and a high-flowability cement mortar was prepared through mechanical mixing. A layered casting process was adopted during the pouring stage: each layer of mortar was cast followed by the placement of one layer of carbon fiber mesh, ensuring full bonding between the fiber mesh and the matrix. After the uniformly mixed mortar was slowly poured into the mold, it was placed on a vibrating table and compacted for 4 min to guarantee the compactness and homogeneity of the material. The specimens, cast after 24 h, were demolded and subsequently transferred to a standard curing room (20 ± 2 °C; relative humidity ≥ 95%) for 28-day standard curing. The experimental procedure is illustrated in Figure 5. The final specimens exhibited a neat appearance and uniform fiber distribution. For each reinforcement ratio, three parallel specimens were prepared to ensure the reliability and repeatability of the test results. The specimen designations are listed in Table 4, which presents the uniaxial tensile test conditions.

### 2.2. Experimental Methods

**Figure 6:**
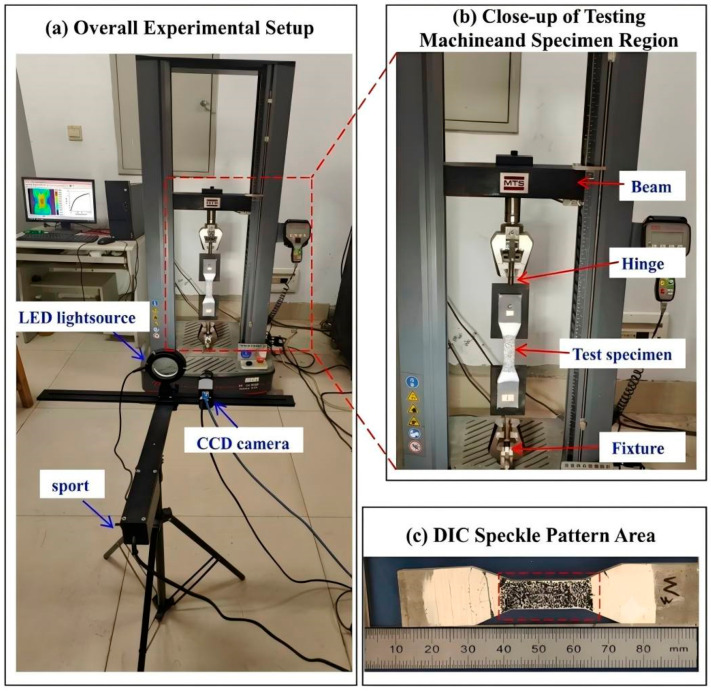
TRC tensile test specimens and instruments. (**a**) Overall Experimental Setup: Shows the electronic universal testing machine with illumination, camera, and support system. (**b**) Close-up of Testing Machine and Specimen Region: Highlights the beam, hinge, test specimen, and fixture assembly. (**c**) DIC Speckle Pattern Area: Shows the speckle pattern region used for Digital Image Correlation (DIC) analysis with specimen ID.

To ensure experimental accuracy, the tensile specimens were designed in a standard dumbbell shape, with overall dimensions of 330 mm × 60 mm × 15 mm (length × width × thickness). The gauge area measured 80 mm × 30 mm × 15 mm, as illustrated in Figure 6. The specimens were used to measure the stress–strain relationship of the TRC specimens under tensile loading, thereby evaluating their tensile performance.

The study employed a quasi-static uniaxial tensile test method, conducted using an MTS E43.104 electronic universal testing machine at Yancheng Institute of Technology. The testing machine was equipped with a 10 kN high-precision load cell and a built-in extensometer. The test was performed under displacement control at a loading rate of 0.2 mm/min to ensure stable deformation of the material during the elastic-plastic stage. To analyze the full-field deformation, a speckle pattern for digital image correlation (DIC) was prepared on the specimen surface in advance. High-resolution industrial camera systems were used to simultaneously acquire images during the test. Synchronization between the camera and the testing machine’s data acquisition card was achieved via hardware triggering, ensuring precise correspondence between the load-time data and the image sequence, thereby providing a reliable basis for subsequent deformation analysis. The industrial camera model was YVSION OSG230-150UM (YVSION, Shenzhen, China), with an effective pixel count of 2.3 megapixels, an image resolution of 1920 × 1200 pixels, and a frame rate of 5 fps.

To ensure the reliability and repeatability of the uniaxial tensile test, the testing system was checked and adjusted before formal loading. The load cell, displacement acquisition system, and DIC image acquisition system were initialized and synchronized. The specimen was then carefully installed in the upper and lower grips, with its longitudinal axis aligned with the loading axis to minimize eccentric tension. The central gauge region used for DIC analysis was kept away from the gripping ends to avoid local clamping disturbance and to ensure that the measured deformation represented the effective tensile region. Before formal loading, a small seating preload of approximately 0.20 KN was applied to eliminate possible gaps between the specimen, fixture, and loading system [30]. After confirming that no visible eccentricity, slippage, or premature cracking occurred, the load and displacement signals were reset to the initial reference state.

The specimen was subsequently loaded under displacement control at a rate of 0.2 mm/min until failure. During the test, the load, crosshead displacement, and image sequence were recorded synchronously. The DIC system continuously captured the surface strain field in the central gauge region, while the load–displacement curve was used to identify the elastic stage, first cracking, multiple-cracking development, and final failure. The validity of the test was evaluated by checking the initial linear segment of the load–displacement curve and by comparing the load drops with the crack initiation and propagation observed from the DIC strain contours. Tests showing obvious end slip, premature gripping failure, or severe out-of-plane bending were excluded. This procedure ensured that the measured mechanical response and full-field strain evolution could be reliably used for evaluating the tensile behavior of TRC and validating the finite element model.

## 3. Model Development

To further reveal the mechanical response and damage evolution of TRC under uniaxial tensile loading, an analytical model incorporating finite element simulation was developed to capture the fiber-matrix interfacial characteristics. This model focuses on simulating the complete behavioral process of the material, ranging from the initial elastic stage, through the development of multiple cracking, to eventual failure. The predictive capability of the model was validated through a systematic comparison between the simulation results and experimental data.

### 3.1. Constitutive Relations and Boundary Conditions

#### 3.1.1. Constitutive Relations

**Figure 7:**
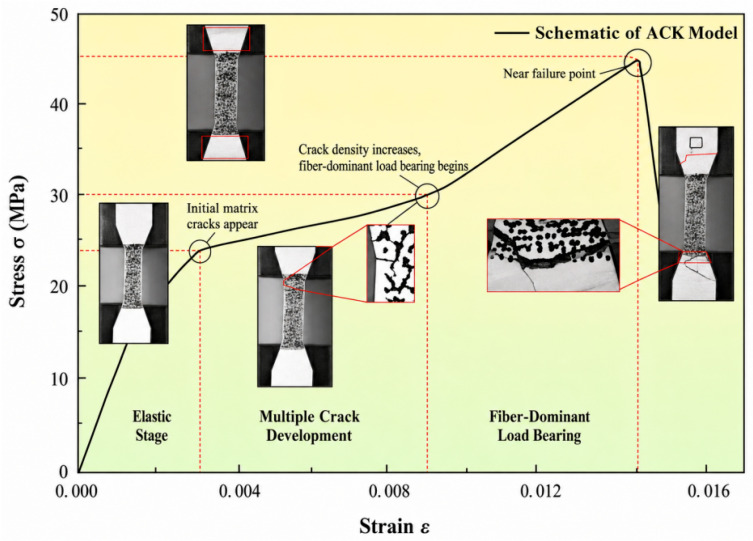
Schematic diagram of the ACK model.

The ACK model, proposed by British scholars Aveston, Cooper, and Kelly [9], was developed to describe the multiple cracking behavior of fiber-reinforced cementitious composites. This model divides the tensile constitutive behavior of TRC (as shown in Figure 7) into three stages and is based on the following assumptions:

(1) The fiber volume fraction must exceed the critical volume fraction;
(2) The tensile stress sustained by the fibers is considerably higher than the tensile strength of the matrix;
(3) The bond between the matrix and the fiber bundles is relatively weak;
(4) Once debonding occurs between the matrix and the fiber bundles, the interfacial bond stress vanishes and is replaced by a constant frictional stress;
(5) The frictional stress at the debonded interface is constant.

The first stage is the elastic stage. In this stage, the elastic modulus of the TRC follows the rule of mixtures, given by:

(1) $$E_{I}=E_{m}V_{m}+E_{f}V_{f}$$

where *E_m_* and *E_f_* are the elastic moduli of the matrix and the fibers, respectively; *V_m_* and *V_f_* are the volume fractions of the matrix and the fibers, respectively. The cracking strain and cracking stress in this stage are given by:

(2) $$\varepsilon_{I}={\left(\frac{12{\gamma_{m}\tau }_{m}E_{f}V_{f}^{2}}{E_{I}V_{m}rE_{m}^{2}}\right)}^{\frac{1}{3}}$$

(3) $$\sigma_{I}=E_{I}\varepsilon_{I}$$

where $\gamma_{m}$ is the toughness of the matrix; r is the equivalent radius of the fiber bundle; $\tau_{m}$ is the shear strength of the matrix.

(4) $$\tau =\frac{rV_{m}\sigma_{\mathrm{mu}}}{2V_{f}x}$$

where $\sigma_{\mathrm{mu}}$ is the tensile strength of the matrix; x is the crack spacing.

The second stage is the multiple cracking stage. The modulus in this stage is zero. When the last crack appears, the corresponding strain is:

(5) $$\varepsilon_{II}=\left(1+0.667\frac{E_{m}V_{m}}{E_{f}V_{f}}\right)\varepsilon_{I}$$

The third stage is the (work-)hardening stage. After the matrix fractures, the load is mainly carried by the fibers. At the end of this stage, the modulus, strain, and stress are, respectively:

(6) $$E_{\mathrm{III}}=E_{f}V_{f}$$

(7) $$\varepsilon_{III}=\left(\varepsilon_{fu}-0.341\frac{E_{m}V_{m}}{E_{f}V_{f}}\varepsilon_{I}\right)$$

(8) $$\sigma_{\mathrm{III}}=\sigma_{\mathrm{II}}+E_{\mathrm{III}}(\varepsilon_{\mathrm{III}}-\varepsilon_{\mathrm{II}})$$

where *ε_fu_* is the ultimate tensile strain of the fiber.

#### 3.1.2. Boundary Conditions

The boundary conditions of the finite element model were defined not only according to the uniaxial tensile test setup, but also in accordance with the basic assumptions of the ACK framework described above. In the ACK model, the tensile response of TRC is simplified as an axial-tension-dominated process: the matrix and fibers first deform compatibly, the matrix subsequently cracks, and the tensile load is then progressively transferred to the fiber bundles through interfacial shear and friction. Therefore, the boundary conditions of the numerical model should minimize additional bending and shear effects and reproduce an approximately uniform axial tensile field in the central gauge region. This treatment is associated with the first two ACK assumptions, namely that the fiber volume fraction should be sufficient to trigger multiple cracking and that the fibers should be capable of sustaining tensile stresses much higher than the cracking strength of the matrix. Accordingly, displacement-controlled axial loading was adopted to ensure a stable transition from the elastic stage to the multiple-cracking stage and finally to the fiber-dominated stage.

To accurately replicate the mechanical environment of the uniaxial tensile test, the boundary conditions of the finite element model were set in accordance with the actual experimental setup. The left end of the model was constrained with a fixed support to simulate the fixed clamp of the testing machine, restricting all six degrees of freedom. The right end was connected to a reference point through a coupling constraint applied to the cross-sectional nodes. A displacement load was then applied at this reference point along the axial direction of the specimen (X-direction) at a loading rate of 0.2 mm/min, consistent with the experimental settings, in order to simulate the quasi-static tensile process.

The above boundary treatment is also associated with the third to fifth ACK assumptions concerning fiber–matrix interfacial behavior. Since the ACK model assumes that the fiber–matrix bond is relatively weak and that, after debonding, the original bond stress is replaced by an approximately constant frictional stress, the numerical model did not impose a perfectly bonded constraint between the fiber bundles and the matrix. Instead, relative slip and stress redistribution were allowed through the interface constitutive law described in Section 3.2. The imposed axial displacement therefore activates matrix cracking, interfacial debonding/slip, and fiber bridging in sequence, which corresponds to the three-stage tensile response predicted by the ACK model. These boundary conditions provide a reasonable basis for analyzing the full-range response of TRC from elastic deformation to multiple cracking and final failure, while avoiding additional deformation modes that are outside the basic assumptions of the ACK framework.

### 3.2. Model Construction

Based on the aforementioned constitutive relations and boundary conditions, a three-dimensional mesoscale finite element model of the TRC specimen was established. The geometric dimensions of the model are consistent with the dumbbell-shaped specimen used in the experiment, with a total length of 330 mm and a central gauge section measuring 80 mm × 30 mm × 15 mm (length × height × width). A separate modeling approach was adopted to individually construct the concrete matrix, the carbon fiber grid, and the interfacial layer between them, thereby accurately characterizing the interactions among the constituent materials.

The concrete matrix was discretized using eight-node reduced-integration solid elements (C3D8R), with its constitutive behavior described by the concrete damaged plasticity (CDP) model. The compressive strength and elastic modulus were determined based on the mix proportion calculations, while the tensile strength was calibrated via splitting tensile test results. The carbon fiber grid was simulated using two-node three-dimensional truss elements (T3D2), which only carry axial loads and can accurately reflect the mechanical characteristics of the fiber bundles. The fiber material was assumed to be linear elastic up to failure, with parameters such as strength and modulus directly taken from the measured values in Table 3.

Interfacial behavior is a key factor influencing the mechanical performance of TRC. Interface elements were assigned in the embedded regions between the fiber bundles and the matrix, modeled using spring elements that account for a bond-slip constitutive law. Key parameters of the interface model, including the initial stiffness, ultimate bond strength, and residual frictional stress, were inversely determined from single-fiber pullout test results, thereby ensuring the fidelity of the interfacial mechanical behavior. The pullout response was characterized by three typical stages: an initial nearly linear ascending stage, a debonding stage after the peak pullout load, and a residual frictional sliding stage. Accordingly, the initial slope of the pullout load–slip curve was used to determine the initial interfacial stiffness, the peak pullout resistance was used to calibrate the ultimate bond strength, and the relatively stable post-peak resistance was used to define the residual frictional stress. These pullout-derived parameters were then introduced into the spring-type interface elements to describe the bond–slip response between the carbon-fiber bundles and the cementitious matrix.

The above modeling strategy was established by translating the basic assumptions of the ACK theory into finite element idealizations. The first ACK assumption, namely that the fiber volume fraction should exceed the critical value required for multiple cracking, was reflected by explicitly assigning different reinforcement ratios through the cross-sectional area and number of carbon-fiber grid layers. Specimens with low reinforcement ratio were retained in the model not because they fully satisfy the ACK multiple-cracking condition, but because they provide a useful comparison for identifying the transition from localized single-crack failure to stable multiple-crack development. The second assumption, that the fibers can sustain tensile stresses much higher than the tensile strength of the matrix, was implemented by using linear-elastic truss elements for the carbon-fiber bundles, while the cementitious matrix was modeled using a damage-plasticity formulation capable of representing tensile cracking and stiffness degradation. This allows the load to be gradually transferred from the cracked matrix to the fiber bundles after matrix cracking.

The third, fourth, and fifth assumptions of the ACK model were mainly introduced through the interfacial constitutive relationship. A perfect bond was not assumed between the carbon-fiber bundles and the cementitious matrix. Instead, spring-type interface elements governed by a bond–slip law were adopted to describe the initial bond stiffness, debonding process, and residual frictional load transfer. Before debonding, the interface transfers shear stress through bond stiffness; after the bond strength is reached, the interfacial stress progressively decreases and approaches a residual frictional level. This residual value represents the constant frictional stress assumed in the ACK model at the debonded fiber–matrix interface. Therefore, the interface parameters directly influence the stress recovery length, crack spacing, and the stability of multiple cracking. In this sense, the finite element model serves as a numerical implementation and extension of the ACK-based analytical framework rather than an independent empirical fitting tool.

**Figure 8:**
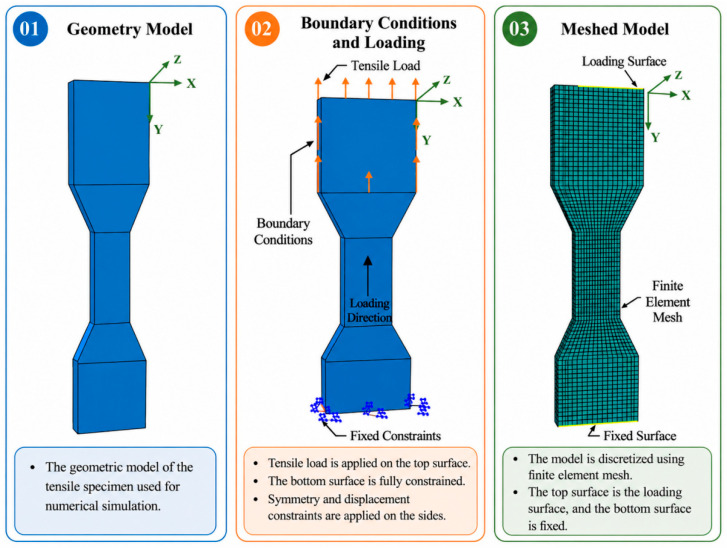
Three-dimensional finite element model of TRC specimen.

During mesh generation, the central gauge section of the specimen was locally refined, with the element size controlled within 2 mm to ensure computational accuracy for crack initiation and propagation. The final model contains approximately 85,000 elements, balancing computational accuracy with solution efficiency. The established finite element model is shown in Figure 8. By systematically incorporating material nonlinearity, geometric nonlinearity, and interfacial behavior, this model provides a reliable numerical analysis platform for subsequently revealing the tensile failure mechanism of TRC.

It should be noted that the proposed finite element model inherits several idealizations from the ACK framework. The gauge region is assumed to be mainly subjected to axial tension, while accidental eccentricity, specimen warping, local gripping damage, and end pull-out are not explicitly considered. Therefore, compared with the experimental results, the numerical prediction may show relatively larger deviations in the initial elastic stiffness and first-cracking load, where the response is sensitive to specimen preparation, gripping conditions, and local matrix defects. In contrast, after matrix cracking, the simulated load–displacement response, crack spacing evolution, and failure mode transition are in better agreement with the experiments, because these responses are mainly governed by fiber bridging and interfacial frictional load transfer, which are explicitly incorporated in the model. In addition, the fiber bundles are simplified as linear-elastic truss elements, and the interface is described by an equivalent bond–slip law with residual frictional stress. As a result, the model is more reliable for predicting the global tensile response and average crack spacing than for resolving highly localized crack opening, individual filament rupture, yarn waviness, or spatial variations in interfacial friction.

Accordingly, the applicability of the proposed approach should be understood within the conditions required by the ACK assumptions. The model is suitable for quasi-static uniaxial tensile analysis of TRC specimens in which the reinforcement ratio is close to or higher than the critical value for multiple cracking, the textile reinforcement remains the primary tensile load-bearing phase after matrix cracking, and the fiber–matrix interface is governed mainly by debonding and frictional slip rather than by complete anchorage or sudden brittle interfacial fracture. The approach is particularly appropriate for comparing the effects of reinforcement ratio, textile orientation, and interfacial treatment on the global tensile response, crack spacing evolution, and failure mode transition of carbon-fiber TRC. However, direct application to TRC systems with very low reinforcement ratios, strongly three-dimensional textile architectures, severe reinforcement eccentricity, dominant end pull-out, cyclic or dynamic loading, or substantially different interfacial mechanisms requires recalibration of the material and interface parameters and further experimental validation.

It should also be noted that the ACK-based framework has certain limitations. This framework is mainly applicable to quasi-static uniaxial tensile behavior of TRC systems in which the textile provides a continuous load-transfer path and the post-cracking response is governed by fiber bridging, interfacial debonding, and frictional slip. For textile structures with pronounced yarn waviness, strong three-dimensional architecture, nonuniform textile spacing, or significant reinforcement eccentricity, the actual stress-transfer path may deviate from the idealized axial bridging assumption. In addition, when the interface is dominated by strong chemical bonding, brittle interfacial fracture, severe coating nonuniformity, or end pull-out, the simplified friction-based interfacial assumption in the ACK framework may not fully describe the real damage evolution. Therefore, when the framework is extended to different textile architectures, different interfacial treatments, or practical engineering members subjected to flexural–tensile coupling, shear effects, cyclic loading, anchorage constraints, or environmental degradation, the material and interfacial parameters should be recalibrated and further experimental validation is required.

### 3.3. Model Validation and Uncertainty

To further examine the mesh dependency of the finite element results, three representative specimen groups, namely P-N-0.44, P-N-1.33, and B-C-0.88, were selected for mesh convergence analysis. These three groups represent low reinforcement ratio, high reinforcement ratio, and combined textile-configuration/interfacial-treatment conditions, respectively. Three mesh sizes were considered in the central gauge region, namely 4 mm, 2 mm, and 1 mm. The 2 mm mesh corresponds to the adopted model, in which the global mesh seed size was set to 0.05 in the model unit and local refinement was applied in the gauge region. The peak load and average crack spacing obtained from different meshes are compared in Table 5.

As shown in Table 5, when the mesh size was reduced from 4 mm to 2 mm, the prediction accuracy of both peak load and crack spacing improved noticeably. However, further refinement from 2 mm to 1 mm produced only limited changes in the numerical results, while the computational cost increased significantly. For all three representative specimen groups, the errors in peak load and crack spacing obtained using the 2 mm mesh remained within approximately 3%. Therefore, the 2 mm mesh was adopted in the following simulations as a balance between numerical accuracy and computational efficiency.

In addition to mesh convergence, the predictive accuracy of the finite element model was further evaluated by comparing the simulated and experimental load–displacement curves at displacement intervals of 1 mm. The relative error was calculated based on the difference between the simulated and experimental load values at the same displacement level. According to the comparison, the error level varied among different specimen groups. For the Control-0 specimen without carbon-fiber reinforcement, the numerical error was generally within ±1–2%. For the uncoated reinforced specimens, including P-N-0.44, P-N-0.88, P-N-1.33, and B-N-0.88, the error was generally within ±2–3%. For the interfacially treated specimens, including P-E-0.88, P-C-0.88, B-E-0.88, and B-C-0.88, the error was generally within ±3–4%. This indicates that the model can reproduce the global tensile response of different specimen groups with acceptable accuracy.

The difference in error level among the three groups is related to the increasing complexity of the load-transfer mechanism. For Control-0, the tensile response is mainly governed by the cracking behavior of the cementitious matrix, and no fiber-bridging or fiber–matrix interfacial slip needs to be considered. Therefore, the numerical prediction is less affected by interfacial uncertainty. For the uncoated reinforced specimens, the model needs to describe matrix cracking, fiber bridging, and frictional stress transfer along the fiber–matrix interface. As a result, the prediction becomes more sensitive to the reinforcement ratio, effective fiber area, fiber modulus, and residual interfacial friction. For the coated specimens, the interface treatment further changes the bond strength, initial interfacial stiffness, and post-debonding frictional resistance, leading to a more complex bond–slip response. Therefore, the numerical error is slightly larger, but it still remains within an acceptable range.

The interfacial properties play a crucial role in the numerical simulation of reinforced specimens. After matrix cracking, the fiber–matrix interface governs the transfer of tensile load from the cracked matrix to the carbon-fiber bundles. Specifically, the initial interfacial stiffness affects the early post-cracking stiffness and load recovery, while the ultimate bond strength controls the initiation of interfacial debonding and the development of subsequent cracks. The residual frictional stress determines the load-transfer capacity after debonding, thereby influencing crack spacing, sawtooth fluctuations in the load–displacement curves, and the stability of multiple cracking. Compared with uncoated specimens, coated specimens exhibit more complex bond–slip behavior due to enhanced bonding and possible spatial nonuniformity of the treated interface. Therefore, interfacial parameters are among the most sensitive inputs in the model, particularly for predicting post-cracking load recovery, crack development, and failure mode transition.

The main sources of parameter uncertainty include the scatter of pullout-derived interfacial parameters, local defects in the cementitious matrix, possible eccentricity during specimen installation, simplification of carbon-fiber bundles as linear truss elements, and the use of an equivalent bond–slip law to represent the complex fiber–matrix interaction. Therefore, the proposed model is more reliable for predicting the global load–displacement response, peak load, average crack spacing, and failure mode transition than for resolving highly localized crack opening, individual filament rupture, or spatial variations in interfacial friction.

## 4. Results and Discussion

### 4.1. Macroscopic Tensile Properties

#### 4.1.1. Load–Displacement Response and Failure Mode Analysis

**Figure 9:**
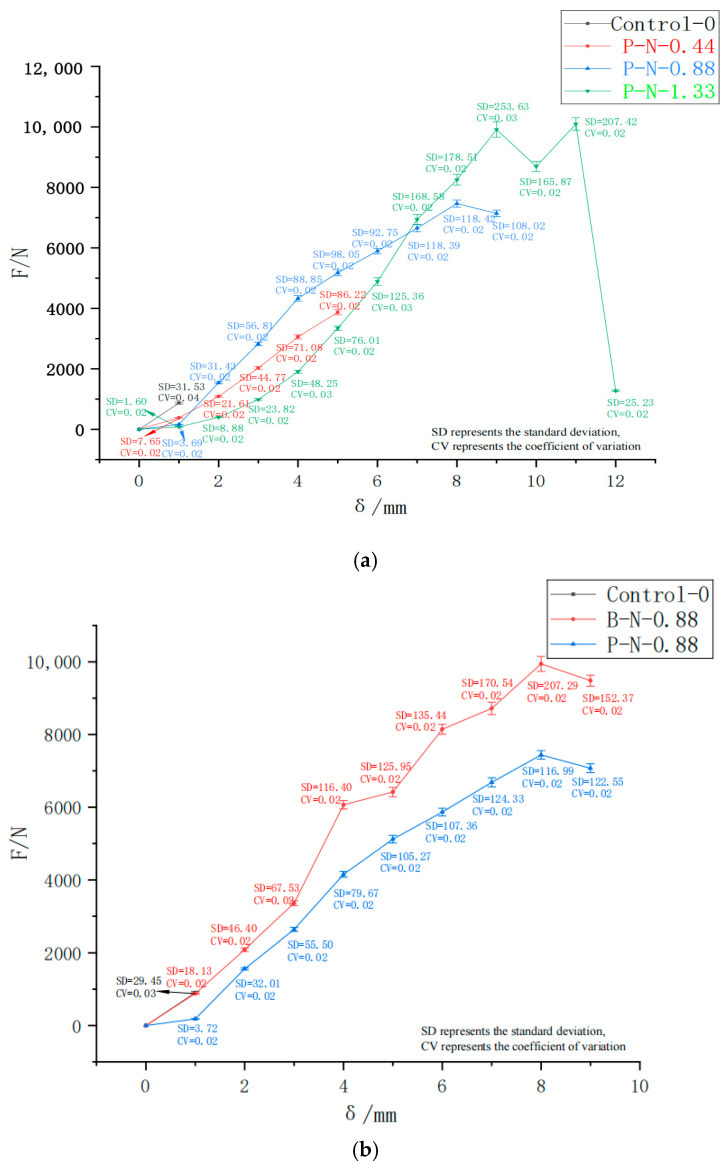

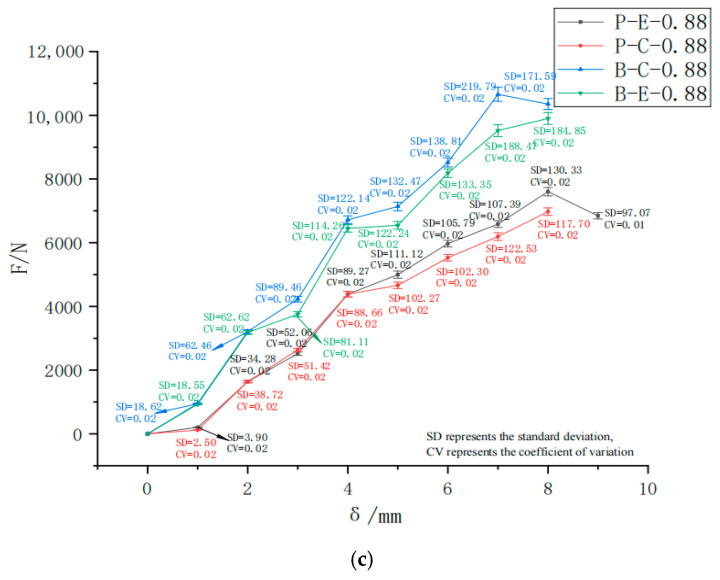
Typical tensile curves of each specimen group. (**a**) Tensile load–displacement curves of TRC specimens with different reinforcement ratios. (**b**) Load–displacement curves of TRC specimens with different reinforcement configurations (0.88% reinforcement ratio). (**c**) Tensile load–displacement curves of 0.88% TRC specimens with different reinforcement configurations and interfacial treatments.

Figure 9 presents the tensile load–displacement responses of TRC specimens under different reinforcement ratios, textile configurations, and interfacial treatments. Each curve represents the average response of three parallel specimens, and the error bars denote the standard deviation (SD) of the measured load at selected displacement levels. The coefficient of variation (CV) was further calculated to evaluate the dispersion of the repeated tests. For statistical plotting, the load values were extracted at displacement intervals of 1 mm from the three repeated tests, and the corresponding mean load, standard deviation, and coefficient of variation at each displacement level were used to construct the load–displacement curves and error bars. As shown in Figure 9, most CV values are within the range of 0.01–0.04, with the majority concentrated around 0.02. This indicates that the repeated tests exhibit good consistency and that the differences among specimen groups are mainly governed by reinforcement ratio, textile configuration, and interfacial treatment rather than experimental scatter.

To further evaluate the statistical significance of the repeated tests, one-way analysis of variance (one-way ANOVA) was performed for each specimen group. The load values of the three parallel specimens were extracted at displacement intervals of 1 mm, and the differences among the three repeated curves within the same condition were statistically examined. The results are summarized in Table 6. For all nine specimen groups, the *p*-values are greater than 0.05, indicating that there is no statistically significant difference among the three repeated specimens under the same test condition. Therefore, the experimental results exhibit good repeatability.

Figure 9a shows the load–displacement curves of TRC specimens with different reinforcement ratios. The Control-0 specimen loses its load-bearing capacity shortly after matrix cracking, showing a typical brittle failure response. In contrast, the reinforced specimens exhibit an evident post-cracking load-bearing stage. As the reinforcement ratio increases from 0.44% to 1.33%, the peak load and ultimate displacement both increase significantly. The P-N-0.44 specimen shows limited post-cracking load growth and relatively early failure, whereas P-N-0.88 exhibits a more stable nonlinear ascending segment. The P-N-1.33 specimen reaches the highest load level and maintains its load-bearing capacity over a larger displacement range. This indicates that increasing the reinforcement ratio improves the fiber-bridging capacity, enhances stress redistribution after matrix cracking, and delays crack localization. The relatively small error bars and low CV values in Figure 9a further confirm the statistical reliability of this reinforcement ratio-dependent trend.

From the perspective of the ACK framework, the curves in Figure 9a can be interpreted as three successive stages. In the initial elastic stage, the matrix and fiber bundles deform cooperatively, and the curve is approximately linear. When the matrix cracking threshold is reached, the response enters the multiple-cracking development stage, where local load drops and subsequent rebounds appear. These fluctuations correspond to repeated crack initiation or propagation, local interfacial debonding/slip, and load recovery through fiber bridging and frictional stress transfer. As the reinforcement ratio increases, the post-cracking stage becomes more stable and extends over a larger displacement range. After crack saturation, the load is mainly carried by the fiber bundles, and the specimen enters a fiber-dominated stage. Correspondingly, the crack pattern transitions from a single dominant crack to multiple cracks, and the average crack spacing decreases from 24.3 mm to 8.7 mm, indicating that the increased reinforcement ratio promotes crack refinement by reducing the effective load-transfer length and improving stress redistribution between adjacent cracks [31].

Figure 9b compares the load–displacement responses of specimens with different textile configurations at the same reinforcement ratio of 0.88%. Compared with the Control-0 specimen, both P-N-0.88 and B-N-0.88 exhibit stable post-cracking load-bearing capacity, confirming that textile reinforcement effectively suppresses brittle matrix failure. Further comparison shows that the B-N-0.88 curve is consistently higher than the P-N-0.88 curve over most of the loading process, with a greater post-cracking load growth rate and a higher peak load. This indicates that the B configuration provides a more effective load-transfer path and a stronger fiber-bridging contribution under the present loading condition. The low CV values, mostly around 0.02, demonstrate that the difference between the two configurations is repeatable and not caused by excessive experimental dispersion.

Figure 9c further compares the influence of interfacial treatment under the same reinforcement ratio of 0.88%. Four treated groups, namely P-E-0.88, P-C-0.88, B-C-0.88, and B-E-0.88, are compared. All four curves exhibit an initial approximately linear stage followed by nonlinear post-cracking development, indicating that the treated interfaces can support fiber bridging and interfacial load transfer after matrix cracking. Overall, the B-series specimens show higher load-bearing capacity than the P-series specimens, suggesting that the effectiveness of interfacial treatment is closely coupled with textile configuration. In the P configuration, P-E-0.88 shows a higher load level than P-C-0.88, indicating that epoxy treatment is more effective than carbon-glue treatment in improving post-cracking load transfer for this configuration. In the B configuration, both B-C-0.88 and B-E-0.88 exhibit relatively high load levels, with B-C-0.88 showing the most pronounced improvement in peak load. This suggests that carbon-glue treatment can more effectively enhance interfacial bonding and fiber-bridging efficiency when combined with the B configuration.

The statistical information in Figure 9c further supports the reliability of the above comparison. Although the absolute SD values increase at higher load levels, the corresponding CV values remain low, generally around 0.02. This indicates that the increase in SD is mainly associated with the higher load magnitude rather than poor repeatability. Therefore, the differences in peak load and post-cracking response among P-E-0.88, P-C-0.88, B-C-0.88, and B-E-0.88 can be considered mechanically meaningful. Overall, Figure 9 demonstrates that the reinforcement ratio primarily governs the stability and extent of the multiple-cracking stage, the textile configuration affects the efficiency of post-cracking load transfer, and the interfacial treatment further modifies the bridging contribution and failure evolution. These three factors jointly control the tensile response of TRC by regulating crack development capacity, fiber-dominated load contribution, and interfacial load-transfer efficiency.

#### 4.1.2. DIC-Based Strain Analysis

**Figure 10:**
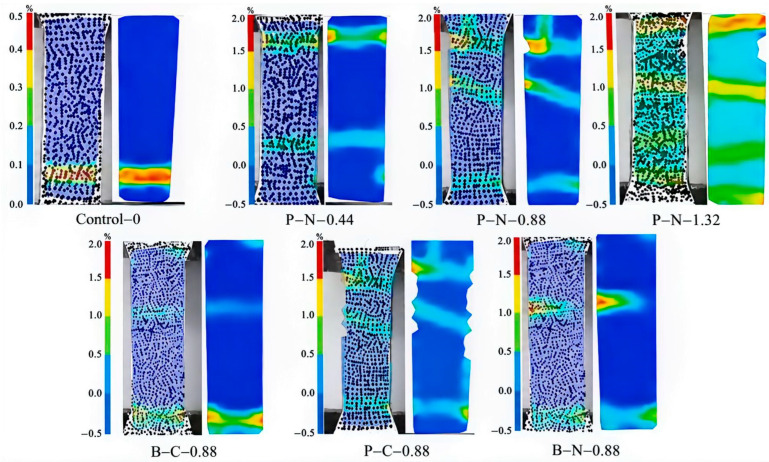
DIC strain distribution contours.

Digital image correlation (DIC) was used to reveal the full-field strain evolution and crack propagation characteristics of TRC specimens under uniaxial tension. Different from the macroscopic load–displacement curves, DIC provides spatial information on strain localization, crack initiation, and post-cracking deformation redistribution. Therefore, the DIC results were interpreted together with the load–displacement response to clarify the relationship between local crack development and global mechanical behavior. Figure 10 presents the full-field strain evolution and crack propagation characteristics of TRC specimens under uniaxial tension, as obtained using digital image correlation (DIC).

For the Control-0 specimen, the strain rapidly concentrated in the middle region of the specimen, forming a narrow localized high-strain band. This indicates that the plain matrix lacked an effective bridging mechanism after cracking, and the first dominant crack rapidly developed into unstable brittle failure. This observation is consistent with the load–displacement curve, where the Control-0 specimen lost its load-bearing capacity shortly after matrix cracking.

For the P-N series, increasing the reinforcement ratio changed the strain field from localized concentration to more distributed deformation. In P-N-0.44, the high-strain region remained concentrated, indicating insufficient crack-arresting capacity. In contrast, P-N-0.88 and especially P-N-1.33 exhibited more dispersed high-strain zones, suggesting that more potential cracking sites were activated and that stress redistribution became more effective. This agrees with the reduction in average crack spacing from 24.3 mm to 8.7 mm and the more stable post-cracking response shown in Figure 9a.

The influence of textile configuration and interfacial treatment can also be identified from the DIC strain contours. Compared with P-N-0.88, B-N-0.88 showed a more distributed strain field, which corresponds to its higher post-cracking load level in Figure 9b. This indicates that the B configuration provides a more effective load-transfer path and improves fiber-bridging efficiency. Similarly, B-C-0.88 exhibited more gradual strain localization than P-C-0.88, suggesting that carbon-glue treatment is more effective when combined with a favorable textile configuration.

Overall, the DIC results establish a direct correspondence between strain localization, crack propagation, and load–displacement response. The formation of a localized high-strain band corresponds to crack initiation and load drop, while the development of multiple high-strain zones corresponds to repeated cracking, fiber bridging, and load recovery. Thus, DIC provides key evidence for explaining the transition from brittle single-crack failure to stable multiple-crack failure in TRC.

#### 4.1.3. Numerical Simulation-Based Strain Analysis

**Figure 11:**
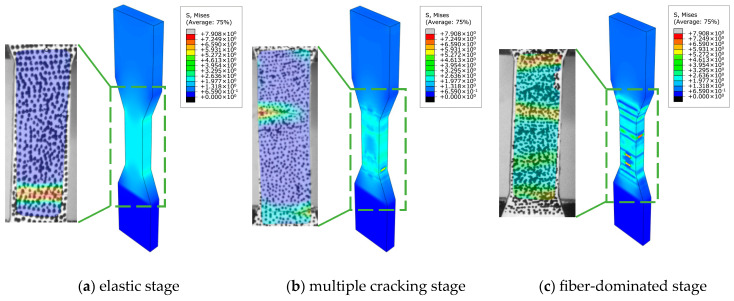
Stress Cloud Diagram.

Based on the established model, this study further analyzed the strain distribution of TRC specimens with different reinforcement configurations and reinforcement ratios through numerical simulation. The simulation results indicate that the reinforcement ratio and configuration have a significant influence on the deformation process, crack propagation, and load-bearing capacity of TRC specimens. As shown in Figure 11, the strain distribution obtained from the simulation is in good agreement with the experimental results, validating the typical three-stage mechanical behavior exhibited by specimens with different configurations during tensile loading, namely the elastic stage, the multiple cracking stage, and the fiber-dominated stage.

At Stage I (elastic stage), the numerical simulation results indicate that, as the reinforcement ratio increases, both the initial stiffness and load-bearing capacity of the specimens are significantly enhanced. In low-reinforcement-ratio specimens (e.g., P-N-0.44), the strain is primarily concentrated during the initial deformation phase, and crack initiation in this stage is relatively difficult. In contrast, high-reinforcement-ratio specimens (e.g., P-N-1.33) exhibit a more uniform strain distribution, with both the initial stiffness and the load-bearing capacity at the same displacement level effectively improved. The simulation demonstrates that an increase in the reinforcement ratio effectively enhances the cooperative load-transfer mechanism among fibers, thereby enabling the specimen to exhibit greater elastic deformation capacity even at relatively low load levels.

Upon entering Stage II (multiple cracking stage), the simulation results show that cracks begin to initiate and propagate, and the strain distribution exhibits pronounced localization. In low-reinforcement-ratio specimens (e.g., P-N-0.44), crack propagation is relatively concentrated, leading to a rapid increase in strain values and early local instability. In high-reinforcement-ratio specimens (e.g., P-N-1.33 and B-N-0.88), however, crack propagation proceeds more gradually, and the specimens display a more uniform strain distribution. This indicates that the combined effects of fiber bridging and interfacial friction effectively delay crack localization and rapid propagation. Notably, in specimens B-N-0.88 and B-C-0.88, the crack distribution is more uniform and the strain field exhibits greater stability, further demonstrating the advantages of longitudinal reinforcement configuration in terms of stress redistribution and crack propagation control.

At Stage III (fiber-dominated stage), the numerical simulation further reveals that once cracks have propagated to a certain extent, the load-bearing capacity of the specimen is primarily governed by the fibers. At this stage, the cracks approach saturation, and the load-bearing capacity is mainly determined by the fiber bridging effect. The simulation results indicate that specimens with a high reinforcement ratio and longitudinal configuration exhibit higher residual load-bearing capacity and lower strain values at this stage. This is consistent with the experimental observations, confirming that a high reinforcement ratio and longitudinal reinforcement configuration effectively enhance the fiber-dominated load-bearing mechanism and significantly improve both the strain distribution and load-bearing capacity during the post-cracking stage. In contrast, low-reinforcement-ratio specimens (e.g., P-N-0.44) experience a more rapid decline in load-bearing capacity during the post-cracking stage, accompanied by larger strains and faster structural instability. In addition, the simulation reveals the significant influence of interfacial treatment on the post-cracking behavior of TRC specimens. In particular, for specimen B-C-0.88, the carbon-epoxy interfacial treatment substantially improved the bond strength between the fibers and the matrix, resulting in a more uniform strain distribution and a further enhancement of the load-bearing capacity during the post-cracking stage. The simulation results suggest that the interfacial treatment, by improving the load-transfer efficiency between the fibers and the matrix, plays a critical supporting role when cracks approach saturation, effectively retarding further crack propagation. By comparison, although specimen P-C-0.88 also received interfacial treatment, the modification effect was relatively limited due to the inherent characteristics of the transverse reinforcement configuration. The interfacial treatment did not significantly alter the crack propagation control mode, indicating that the effectiveness of interfacial treatment is constrained under transverse reinforcement configurations.

In summary, the model-based strain analysis validates the crack propagation patterns and load-bearing capacity trends observed in the experimental results. The simulation results demonstrate that a high reinforcement ratio and longitudinal configuration can effectively control crack development and enhance the post-cracking load-bearing capacity and toughness of the specimens. Furthermore, interfacial treatment, particularly when combined with a longitudinal configuration, can significantly improve the fiber-matrix bond strength, thereby enhancing the load-transfer efficiency during the post-cracking stage.

### 4.2. Mechanism Analysis

Based on the macroscopic load–displacement response, DIC full-field strain evolution, and finite element simulation, the tensile failure process of carbon-fiber TRC can be interpreted as a coupled damage evolution process governed by matrix cracking, interfacial load transfer, stress recovery, and fiber bridging. From the viewpoint of fracture mechanics, the transition from single-crack brittle failure to stable multiple cracking is essentially controlled by the balance between the strain energy released by matrix cracking and the energy dissipated through crack surface formation, interfacial debonding, frictional slip, and fiber bridging. In the initial elastic stage, the matrix and carbon-fiber bundles deform compatibly. Once the local tensile stress in the weakest matrix section reaches the matrix cracking strength, the first crack initiates, leading to a sudden release of local strain energy and a load drop in the macroscopic curve. At this moment, the tensile load originally carried by the cracked matrix is transferred to the fiber bundles across the crack plane and is then redistributed back to the surrounding matrix through interfacial shear and residual friction.

After the first crack forms, a stress recovery zone is generated on both sides of the crack. Within this zone, the matrix stress gradually increases from nearly zero at the crack plane to the remote tensile stress level through fiber–matrix interfacial load transfer. A subsequent crack can only initiate when the recovered matrix stress again reaches the matrix cracking strength outside the damaged zone of the previous crack. Therefore, crack spacing is not only a geometric result observed after testing, but also a mechanical consequence of the load-transfer length. Previous analytical studies have shown that the final crack spacing of TRC is closely related to the load-transfer length, which also governs crack width evolution. In the present study, increasing the reinforcement ratio from 0.44% to 1.33% reduced the average crack spacing from 24.3 mm to 8.7 mm, corresponding to a 64% reduction. This indicates that higher fiber content shortens the effective stress recovery length and enables more matrix sections within the gauge length to reach the cracking condition. As a result, the released cracking energy is distributed among multiple cracks rather than being concentrated at one dominant fracture plane, which explains the simultaneous increase in ultimate tensile stress from 1.86 MPa to 4.08 MPa and ultimate tensile strain from 0.86% to 1.72%.

The characteristic drop–rebound fluctuations in the load–displacement curves further reveal the sequential nature of crack evolution. Each load drop corresponds to a local damage event, including matrix crack initiation or rapid crack propagation, accompanied by local interfacial debonding and slip. The subsequent rebound reflects the re-establishment of load-bearing capacity through fiber bridging and frictional stress transfer along the debonded interface. Therefore, the sawtooth response is not a random fluctuation of the testing curve, but a macroscopic manifestation of repeated crack nucleation, stress release, interfacial energy dissipation, and load redistribution. This interpretation is supported by the DIC strain contours: localized high-strain bands appear at crack initiation, while increasing reinforcement ratio gradually transforms the strain field from a single localized band into multiple distributed strain concentration zones. Quantitatively, the decrease in the strain inhomogeneity index from 0.65–0.78 to 0.28–0.35 indicates that the deformation becomes more uniformly distributed as the crack system evolves from localized fracture to stable multiple cracking.

The effects of textile orientation and interfacial treatment can be further explained by the efficiency of the load-transfer path. Compared with the transverse configuration, the longitudinal configuration provides a more continuous fiber-bridging path along the tensile direction, allowing the carbon-fiber bundles to participate more effectively in post-cracking load transfer. This increases the ability of the composite to redistribute stress after each cracking event and delays the localization of deformation. As a result, the longitudinal configuration increases the ultimate tensile stress and ultimate tensile strain by 32.7% and 46.2%, respectively. This improvement is associated with the enhancement of interfacial frictional stress by approximately 35–45%, which reduces the stress recovery length and promotes the formation of more closely spaced cracks. Carbon-glue interfacial treatment further strengthens this mechanism by improving fiber-bundle integrity, chemical bonding, and mechanical interlocking at the fiber–matrix interface. Under the longitudinal configuration, this treatment increases the ultimate tensile stress and ultimate tensile strain by 36.9% and 42.1%, respectively, and reduces the average crack spacing to 8.1 mm. These results indicate that the treated interface can sustain higher shear transfer before debonding and maintain more stable residual frictional resistance after debonding, thereby stabilizing crack propagation and shifting the failure mode toward fiber rupture-dominated failure.

The finite element simulation provides further evidence for this mechanism. The model incorporating an explicit bond–slip interface law reproduces the transition from the elastic stage to multiple cracking and then to the fiber-dominated stage, with the peak-load error remaining below 5% and the crack spacing error below 10%. This agreement indicates that the dominant mechanism controlling the tensile response is not only the intrinsic tensile strength of the matrix or fibers, but also the interfacial stress transfer capacity that determines the recovery of matrix stress between adjacent cracks. When the reinforcement ratio is insufficient or the interface is weak, the stress recovery length becomes too large relative to the gauge length, preventing the formation of additional cracks and resulting in brittle single-crack failure. In contrast, when the reinforcement ratio and interfacial frictional resistance are sufficiently high, the matrix can repeatedly undergo cracking, stress recovery, and re-cracking until crack saturation is reached. The material then enters a fiber-dominated stage, in which the final failure depends on the competition between fiber tensile rupture and interfacial pull-out.

Therefore, the tensile failure of carbon-fiber TRC should not be understood as a simple strength-controlled fracture process. Instead, it is a multistage damage evolution process controlled by the coupling of reinforcement ratio, textile orientation, and interfacial load-transfer efficiency. The reinforcement ratio determines whether sufficient bridging capacity is available to activate multiple cracking; the textile orientation determines whether the fiber bundles can form an efficient load-transfer path along the tensile direction; and the interfacial treatment determines whether stress can be effectively transferred and dissipated through debonding and residual friction. These three factors jointly regulate the transition from brittle single-crack failure to quasi-ductile multiple-crack failure, providing a mechanistic basis for the design of carbon-fiber TRC with improved crack control and tensile performance.

The present results provide practical guidance for the design of carbon-fiber TRC by clarifying how reinforcement ratio, textile orientation, and interfacial treatment affect tensile capacity, crack spacing, and failure mode. These findings may support the selection of textile configurations and provide supplementary evidence for TRC or carbon-fiber grid strengthening applications where crack control and corrosion-free reinforcement are required.

## 5. Conclusions

In this study, the tensile failure process of carbon-fiber textile-reinforced concrete (TRC) was investigated through uniaxial tensile tests, DIC full-field strain analysis, and finite element simulation within the ACK framework. The main conclusions are summarized as follows:

(1) The tensile behavior of carbon-fiber TRC is governed by the coupled interactions among reinforcement ratio, textile configuration, and fiber–matrix interfacial properties. Within the ACK theoretical framework, the overall response is characterized by a sequential evolution process, including matrix cracking initiation, interfacial debonding and slip, and progressive load transfer to the fiber reinforcement phase, ultimately leading to a fiber-dominated failure mode. This mechanism explains the observed transition from single cracking to stable multiple cracking and crack refinement.
(2) The combined use of uniaxial tensile testing, digital image correlation (DIC), and finite element simulation provides a consistent multiscale framework for interpreting the tensile response of TRC. The experimental observations, full-field strain evolution, and numerical predictions exhibit good agreement in capturing crack initiation, propagation behavior, and strain localization, thereby validating the effectiveness of the proposed ACK-based modeling approach.
(3) It should be noted that the present model is established under the idealized assumptions of the ACK framework, including quasi-static loading conditions, axial tensile dominance, and simplified interface bond–slip behavior. Therefore, its applicability is most suitable for TRC systems governed by stable multiple cracking mechanisms under monotonic loading.

Further research is still required to extend and validate the proposed framework under more complex service conditions. In particular, the tensile and interfacial behavior of TRC should be examined under extreme low-temperature or freeze–thaw environments, elevated-temperature or fire exposure, and chloride-rich or wet–dry cyclic conditions. In addition, larger-scale structural members and different textile architectures, coating systems, matrix types, and anchorage conditions should be investigated to improve the applicability of the framework for practical engineering design.

## Figures and Tables

**Table 1:** Mix design of the matrix.

	Binder	Fine Aggregate		Chemical Admixture			
Matrix material	Ordinary Portland Cement	Natural river sand	Marble powder	UEA expansive agent	Accelerator	HPMC (hydroxypropyl methyl cellulose) water retention agent	Water
Mass fraction	21.0%	36.9%	26.3%	0.5%	0.5%	0.5%	14.3%

**Table 2:** Chemical composition of the cement.

Types	Chemical Composition/%								
	SiO_2_	Al_2_O_3_	Fe_2_O_3_	MgO	CaO	SO_3_	R_2_O	MnO	H_2_O
Cement	20.12	5.51	3.95	1.81	62.31	2.62	0.52	0	0

**Table 3:** Properties of Carbon Fiber Fabric.

	Tensile Strength, MPa	Tensile Modulus of Elasticity, GPa	Elongation at Break, %
Warp direction	3.16 × 10^3^	219	1.3
Weft direction	2.58 × 10^3^	319	1.2

**Table 4:** Uniaxial Tensile Test Matrix.

Specimen Number	Load-Bearing Fiber Weaving Method	Hardener	Reinforcement Ratio
Control-0	None	None	0%
P-N-0.44	Plain weave	None	0.44%
P-N-0.88		None	0.88%
P-N-1.33		None	1.33%
P-E-0.88		epoxy resin	0.88%
P-C-0.88		Carbon fiber impregnation resin	0.88%
B-N-0.88	Woven Mesh	None	0.88%
B-E-0.88		epoxy resin	0.88%
B-C-0.88		Carbon fiber impregnation resin	0.88%

Note: P/B: Weaving method (P-plain weave, B-braided); N/E/C: Curing agent type (N-none, E-epoxy resin, C-carbon glue); Numeric value: reinforcement ratio in percentage.

**Table 5:** Mesh dependency analysis of the finite element model based on three representative specimen groups.

Specimen Group	Mesh Size in Gauge Region	Peak Load/N	Error in Peak Load	Remark
P-N-0.44	4 mm	4185	2.67%	Coarse mesh
	2 mm	4268	0.74%	Adopted mesh
	1 mm	4320	0.47%	Fine mesh
P-N-1.33	4 mm	9850	3.43%	Coarse mesh
	2 mm	10,140	0.59%	Adopted mesh
	1 mm	10,265	0.64%	Fine mesh
B-C-0.88	4 mm	10,620	3.45%	Coarse mesh
	2 mm	10,960	0.36%	Adopted mesh
	1 mm	11,035	0.32%	Fine mesh

**Table 6:** Statistical significance analysis of repeated specimens under each test condition.

Specimen Group	Specimen Count	Statistical Method	*p*-Value	Significance Result
Control-0	3	One-way ANOVA	*p* > 0.05	Not significant
P-N-0.44	3	One-way ANOVA	*p* > 0.05	Not significant
P-N-0.88	3	One-way ANOVA	*p* > 0.05	Not significant
P-N-1.33	3	One-way ANOVA	*p* > 0.05	Not significant
P-E-0.88	3	One-way ANOVA	*p* > 0.05	Not significant
P-C-0.88	3	One-way ANOVA	*p* > 0.05	Not significant
B-N-0.88	3	One-way ANOVA	*p* > 0.05	Not significant
B-E-0.88	3	One-way ANOVA	*p* > 0.05	Not significant
B-C-0.88	3	One-way ANOVA	*p* > 0.05	Not significant

## Data Availability

The original data presented in this study are included in the paper. For further inquiries, please contact the corresponding author.

## References

[1] Luo Y., Lin G., Chen J.-F., Wu J. (2025). Thermoplastic fiber-reinforced polymer composites in construction: A state-of-the-art review. Case Stud. Constr. Mater..

[2] Brameshuber W. (2006). Textile Reinforced Concrete—State-of-the-Art Report of RILEM TC 201-TRC.

[3] Williams Portal N., Lundgren K., Wallbaum H., Malaga K. (2015). Sustainable potential of textile-reinforced concrete. J. Mater. Civ. Eng..

[4] Hegger J., Voss S. (2008). Investigations on the bearing behaviour and application potential of textile reinforced concrete. Eng. Struct..

[5] Promis G., Gabor A., Maddaluno G., Hamelin P. (2010). Behaviour of beams made of textile reinforced mineral matrix composites. Compos. Struct..

[6] Wu Z., Deng M., Qiu Z., Li T., Dong Z. (2025). Coupling effect of short-fiber kind and matrix strength on uniaxial tensile behavior of textile-reinforced high-ductility concrete (TRHDC). Arch. Civ. Mech. Eng..

[7] Dinh N.H., Pham H.H., Kim S.-H., Choi K.-K. (2024). Tensile Performance and Cracking Characteristics of Sustainable Textile-Reinforced Cementitious Composites Utilizing Expanded Glass Aggregate and Fly Ash Replacement. Constr. Build. Mater..

[8] Hartig J., Häußler-Combe U., Schicktanz K. (2008). Influence of bond properties on the tensile behaviour of Textile Reinforced Concrete. Cem. Concr. Compos..

[9] Aveston J., Kelly A. (1973). Theory of multiple fracture of fibrous composites. J. Mater. Sci..

[10] Peled A., Bentur A. (2003). Fabric structure and its reinforcing efficiency in textile reinforced cement composites. Compos. Part A Appl. Sci. Manuf..

[11] Mobasher B., Pahilajani J., Peled A. (2006). Analytical simulation of tensile response of fabric reinforced cement based composites. Cem. Concr. Compos..

[12] Dahlhoff A., Raupach M. (2023). Crack Analysis of Textile Reinforced Concrete Using Automated Crack Evaluation via Digital Image Correlation. Buildings.

[13] Zhao J., Sobczyk M., Liebscher M., Ahmed A.H., Wallmersperger T., Mechtcherine V. (2025). Numerical and experimental assessment of thermally exposed textile-reinforced alkali-activated concrete with altered impregnated interphases. Cem. Concr. Compos..

[14] Li B., Xiong H., Jiang J., Dou X. (2019). Tensile behavior of basalt textile grid reinforced ECC. Compos. Struct..

[15] Jia M., Wei F., Xie W., Jia Y., Li Y., Zheng H. (2025). High-performance fiber- and textile-reinforced concrete composite materials for use in construction applications: A review. J. Text. Inst..

[16] D’Antino T., Papanicolaou C. (2017). Mechanical characterization of textile-reinforced inorganic-matrix composites. Compos. Part B Eng..

[17] Zhu D., Li X., Li A. (2022). Influence of fiber volume fraction in warp and weft directions on tensile properties of AR-glass TRC. Acta Mater. Compos. Sin..

[18] Vandereecken G., Van Hemelrijck D., García Mayo S., Seveno D., Tysmans T. (2026). Impact of crimp and 3D textile architecture on woven basalt Textile Reinforced Cement (TRC) composites flexural performance. Constr. Build. Mater..

[19] Paul S.C., van Zijl G.P.A.G., Šavija B. (2018). Influence of textile geometry on tensile behaviour of TRC. Mater. Des..

[20] Messori M., Nobili A., Signorini C., Sola A. (2018). Mechanical performance of epoxy-coated AR-glass fabric Textile Reinforced Mortar: Influence of coating thickness and formulation. Compos. Part B Eng..

[21] Donnini J., Corinaldesi V., Nanni A. (2016). Mechanical properties of FRCM using carbon fabrics with different coating treatments. Compos. Part B Eng..

[22] Elseady A.A.E., Zhuge Y., Ma X., Chow C.W.K., Lee I., Zeng J. (2026). Effect of textile configuration and matrix composition on the self-sensing performance of carbon fibre textile-reinforced cementitious composites under flexural loading. Structures.

[23] Mobasher B., Stang H., Shah S.P. (1990). Microcracking in fiber reinforced concrete. Cem. Concr. Res..

[24] Wu C., Pan Y., Yan L. (2023). Mechanical properties and durability of Textile Reinforced Concrete (TRC)—A review. Polymers.

[25] Yin S., Xu S., Li H. (2013). Improved mechanical properties of textile reinforced concrete thin plate. J. Wuhan Univ. Technol..

[26] Mumenya S.W., Tait R.B., Alexander M.G. (2011). Evaluation of toughness of textile concrete. Mater. Struct..

[27] Dong Z.-F., Deng M.-K., Zhang C. (2020). Experimental Study on Uniaxial Tensile Performance of Fiber-Reinforced High Ductility Concrete. J. Civ. Eng..

[28] Contamine R., Si Larbi A., Hamelin P. (2011). Contribution to direct tensile testing of textile reinforced concrete (TRC) composites. Mater. Sci. Eng. A.

[29] Hegger J., Will N., Bruckermann O., Voss S. (2006). Load-bearing behaviour and simulation of textile reinforced concrete. Mater. Struct..

[30] Sharifi I., Sherzai M.H., Javanmard A., Yalçındağ F., Yaman İ.Ö. (2025). Uniaxial tensile performance in the warp and weft directions of carbon textile reinforced concretes with two different matrices. Constr. Build. Mater..

[31] Saidi M., Gabor A. (2025). Analytical modelling of the load-transfer length and the crack width evolution of textile-reinforced cementitious (TRC) matrix composites. Compos. Struct..

